# Prevalence of advanced HIV disease in sub-Saharan Africa: a multi-country analysis of nationally representative household surveys

**DOI:** 10.1016/S2214-109X(24)00538-2

**Published:** 2025-02-26

**Authors:** Dominik Stelzle, Ajay Rangaraj, Joseph N Jarvis, Nirina H Razakasoa, George Perrin, Daniel Low-Beer, Meg Doherty, Nathan Ford, Shona Dalal

**Affiliations:** a Global HIV, Hepatitis and STIs Programmes, World Health Organization, Geneva, Switzerland; b Department of Clinical Research, Faculty of Infectious and Tropical Diseases, London School of Hygiene and Tropical Medicine, London, UK; c Botswana Harvard Health Partnership, Gaborone, Botswana; d Regional Office for Africa, World Health Organization, Brazzaville, Republic of the Congo; e Centre for Integrated Data and Epidemiological Research, University of Cape Town, Cape Town, South Africa

## Abstract

**Background:**

Advanced HIV disease (AHD) is a critical stage in the progression of HIV infection and is associated with heightened susceptibility to opportunistic infections, malignancies, and other life-threatening complications. Estimates of the burden of AHD in sub-Saharan Africa are scarce but are needed for programme planning which includes the allocation of resources and the monitoring of outcomes. The aim of the study was to assess the prevalence of and the number of people living with HIV with AHD.

**Methods:**

In this nationally representative study, we analysed data from 13 Population-based HIV Impact Assessment (PHIA) household surveys conducted between 2016 and 2021 to determine the proportion of adults living with HIV who have AHD (defined as CD4 count <200 cells per mm^3^). We analysed the prevalence of AHD by various demographic and socioeconomic factors; we then estimated the number of individuals with AHD in sub-Saharan Africa by combining these proportions with the latest UNAIDS HIV estimates for the region by the treatment and care cascade. We also assessed policies related to the provision of the recommended package of care for the diagnosis and management of AHD.

**Findings:**

A total of 28 040 people living with HIV were included in this study from 13 PHIA surveys. 19 364 were females (weighted percentage 64·5%) and 8676 (35·5%) were males, and the median age of participants was 38 years (IQR 30–47). Pooled across the 13 countries, 9·8% (95% CI 9·3–10·3) had a CD4 cell count of less than 200 cells per mm^3^. AHD was more common among males than females (13·2% *vs* 8·0%) and differed across the treatment cascade: 15·4% among people living with HIV who did not know their HIV status, 20·9% among people who knew their status but were not on antiretroviral treatment (ART), 29·5% among people who were on ART but not virally suppressed, and 4·3% among people who were virally suppressed. Extrapolating these results to sub-Saharan Africa yielded an estimated 1·88 million people living with AHD (uncertainty interval [UI] 1·58–2·20); 920 000 (UI 770 000–1·07 million) females and 970 000 (UI 810 000–1·13 million) males.

**Interpretation:**

Despite advances in ART that have transformed HIV into a manageable chronic condition, a substantial number of people continue to develop AHD. These figures highlight the need for urgent and innovative programmatic improvements in monitoring, prevention, testing, and diagnosis of AHD in the context of well-established and maturing ART programmes.

**Funding:**

None.

## Introduction

HIV remains a persistent global health concern, affecting millions of individuals worldwide. Remarkable advancements in expanding access to antiretroviral treatment (ART) have transformed HIV into a manageable chronic condition. However, a substantial number of people continue to progress to an advanced stage of disease. Advanced HIV disease (AHD) is characterised by a severely compromised immune system as indicated by a CD4 count below 200 cells per mm^3^ or a WHO HIV clinical stage of 3 or 4.^1^ AHD is a critical stage in the progression of HIV infection and is associated with heightened susceptibility to opportunistic infections, malignancies, and other life-threatening complications. As a result, individuals with AHD have higher mortality, even after initiating ART.^2^, ^3^ WHO has published several guidelines on the diagnosis of AHD and preventive treatment of concomitant conditions, and recommends a package of care for patients with AHD.^1^, ^4^, ^5^

Numerous studies have examined the prevalence and risk factors associated with AHD in different countries and settings in sub-Saharan Africa.^6^, ^7^ Delayed diagnosis, inadequate access to ART, medication non-adherence and care interruption, and socioeconomic factors leading to poor ART adherence all play a role in progression to AHD.^6^, ^8^, ^9^ AHD persists globally, yet, an estimation of population level burden of AHD is lacking.

**Research in context**

**Evidence before this study**

We searched PubMed from database inception on April 18, 2024, for the terms “Advanced HIV disease” and “Africa” in the title and abstracts for publications from any year. No language restrictions were applied to the search. A broad range of studies, reviews, and commentaries were found. Reviews often focused on the management of advanced HIV disease or palliative care of patients with advanced HIV disease. Studies presented data on specific populations (such as men who have sex with men, or people who use drugs) and mainly focused on people newly diagnosed with HIV. No study on the burden of advanced HIV disease in sub-Saharan Africa was found.

**Added value of this study**

In this study we estimated the proportion of people living with HIV with advanced HIV disease and the burden of advanced HIV disease in sub-Saharan Africa. We estimated that almost 1·9 million people had advanced HIV disease, despite the progress that has been achieved in reaching the 95–95–95 treatment targets. Advanced HIV disease was more common among males than females, and was increasingly seen in older age groups. The prevalence of advanced HIV disease was highest among people on antiretroviral treatment (ART) who were not virally suppressed; however, almost one in 20 people with suppressed viral loads also had advanced HIV disease.

**Implications of all the available evidence**

Although good progress has been made in providing ART to people living with HIV and keeping people engaged in care, HIV-related deaths have not declined as much as expected. We showed that widespread roll-out of ART will not be enough to prevent advanced HIV disease. Innovative programmatic improvements in the uptake of recommendations from the WHO package of care for advanced HIV disease are crucial for improving patient outcomes and preventing HIV-related deaths.

The aim of this study was to describe the proportion of adults living with HIV who have AHD, defined as a CD4 count of less than 200 cells per mm^3^, identify factors associated with the development of AHD, including socioeconomic, demographic, and health-care related determinants, and estimate the number of individuals with AHD in sub-Saharan Africa. We also aimed to assess policies related to the provision of the recommended package of care for the identification and management of AHD.

## Methods

### Study design and participants

We analysed AHD prevalence in population-based household surveys, estimated the number of people with AHD in sub-Saharan Africa, and assessed country-level policies. For the analysis of AHD prevalence disaggregated by various factors, we used data from 13 publicly available Population-based HIV Impact Assessment surveys (PHIA surveys) which contained information on CD4 testing. For the estimation of the number of people with AHD in sub-Saharan Africa, we used the latest HIV estimates from UNAIDS. We limited the definition of AHD to a CD4 count below 200 cells per mm^3^ because no information was available on clinical staging or opportunistic infections in the surveys, and because the drop in CD4 count tends to occur before the presence of symptoms.^2^ The data used for this study were publicly available, pseudonymised data collected through PHIA surveys; no identifying information was used.

### Procedures

PHIAs are nationally representative household surveys that include questionnaires on several HIV-related indicators and include HIV and CD4 count testing using Pima CD4 point-of-care assays (Abbott, IL, USA).^10^ Sex was self-reported in the survey with options of male or female. Rural and urban location were determined by country census data or official statistics, and wealth was measured through a series of questions on household ownership of various assets and infrastructure.

We included all 13 publicly available PHIA datasets with CD4 cell count testing which were conducted between 2016 and 2021 (appendix p 5). These were conducted in Botswana, Cameroon, Côte d’Ivoire, Eswatini, Ethiopia, Lesotho, Malawi, Mozambique, Namibia, Tanzania, Uganda, Zambia, and Zimbabwe. The quality of the CD4 test was ensured by conducting instrument verification, and comprehensive training and quality control. Across the 13 countries, 28 040 (91·6%) of the 30 609 sampled people living with HIV were tested with the CD4 count point-of-care test and had a valid test result. Slightly more females did not have a valid CD4 count result when compared with males and this was also the case for those from rural areas compared with urban areas. Age did not differ between people with and without a valid CD4 count result (appendix p 5).

UNAIDS produces annual estimates of the number of new HIV infections, HIV prevalence, and the number and the proportion of people on ART for 172 countries.^11^ UNAIDS also reports the proportion of people who know their HIV status and the proportion who are virally suppressed, both regionally and globally. For this analysis, we used estimates for 2022. The following indicators were used: total number of people living with HIV in sub-Saharan Africa, the total number of people on ART, and the proportion and number of people who were virally suppressed (all disaggregated by sex).

The National Commitments and Policy Instrument (NCPI) is an integral component of Global AIDS Monitoring that aims to monitor the development and implementation of policies and laws related to the HIV response including testing, treatment, stigma or discrimination, community leadership, human rights, and surveillance.^12^ Responses to NCPI were provided by national authorities. We analysed the 2023 NCPI to assess the number of countries reporting to have adopted the WHO guidelines recommending a package of interventions to all patients presenting with advanced HIV disease.

### Outcomes

The main outcome measured was advanced HIV disease as defined by a CD4 cell count <200 cells per mm3 among people living with HIV in 13 countries. Prevalence was determined by sex, age group, urban or rural residence, wealth quintile, and the treatment cascade. Policy outcomes included adoption of WHO guidelines, and implementation of the recommended package of care for AHD.

### Statistical analysis

For the analyses of PHIA surveys, we applied individual blood survey weights provided as part of the PHIA data sets (each participants had an individual weight) that consider the complex design of PHIA surveys (enumeration areas as strata and households as clusters) to make results nationally representative; blood weights additionally take into account non-response and a post-stratification factor which adjusts for under-coverage to a set of population projections for the country. In this scenario, each participant's blood weight can be interpreted as the number of individuals that the participant represents in the population who could have participated in blood testing, accounting for selection and non-response of enumeration areas, household, individual, and blood testing. For the estimation of uncertainty around the estimates, 95% CIs derived from Jackknife variance estimations were used.^13^ Data were analysed for each country separately and pooled across all 13 countries (by amending the example R code for individual and pooled country estimates as published in the data use manual).^13^

We assessed the weighted proportion and 95% CI of people with AHD (defined as CD4 count <200 cells per mm^3^) by the following factors: sex, age group (by 5 years), place of residence (urban *vs* rural; in Lesotho, semi-urban was an additional category and was coded as rural for this analysis), wealth quintile, and the testing and treatment cascade (ie, people who do not know their HIV status, people who know their HIV status but who are not on ART, people who are on ART but not virally suppressed, and people who are on ART and virally suppressed). The last category includes people with either undetectable viral load or with a viral load of less than 1000 copies per millilitre. We further examined AHD prevalence by the testing and treatment cascade disaggregated by sex**.**

We assessed the distribution of these factors among people with AHD and their association with AHD prevalence using mixed-effects logistic regression models with the countries included as random effects, and sex, age, area of residence, as well as the categories of the treatment cascade as fixed effects. Mixed-effects models were run with the glmer function of the *lme4* package in R.^14^ The association between the time on ART and AHD was assessed by a χ^2^ test.

To extrapolate the proportion of people with AHD from household surveys to all people living with HIV in sub-Saharan Africa, we multiplied sex-disaggregated AHD proportions for the treatment cascade with the sex-disaggregated number of people living with HIV who were not on ART, who were on ART but not virally suppressed, and who were virally suppressed. For this purpose, treatment cascade categories were combined for people who were unaware of their status and people who knew their status but were not on ART. Uncertainty intervals were derived from the uncertainty intervals around the UNAIDS estimates. We further examined the number of people with AHD for a scenario in which the 95–95–95 treatment targets had already been met.

### Role of the funding source

There was no funding source for this study.

## Results

A total of 28 040 people living with HIV with a CD4 cell count result in 13 PHIA surveys were included in our analysis (table 1). Among these, 19 364 were female (weighted percentage 64·5%) and 8676 (35·5%) were male, the median age was 38 years (IQR 30–47), and 14 558 people (weighted percentage 53·3%) lived in rural areas. Across the 13 countries, 25·8% (n=4762) of people living with HIV were not aware of their status (range: 4·8% in Botswana to 50·2% in Côte d’Ivoire), 4·1% (n=1147) were aware they are living with HIV but were not on ART, 7·6% (n=1871) were on ART but not virally suppressed, and 62·5% (n=20 140) were virally suppressed (range: 33·5% in Côte d’Ivoire to 91·3% in Botswana).

**Table 1:** Baseline characteristics of included participants living with HIV across the 13 Population-based HIV Impact Assessment surveys

		**Botswana (2021)**	**Cameroon (2017)**	**Côte d'Ivoire (2017)**	**Eswatini (2016)**	**Ethiopia (2017)**	**Lesotho (2020)**	**Malawi (2020)**	**Mozambique (2021)**	**Namibia (2017)**	**Tanzania (2016)**	**Uganda (2016)**	**Zambia (2016)**	**Zimbabwe (2020)**	**Overall**
Total sample size		3419	975	436	3000	614	3689	2463	2034	2442	1823	1747	2446	2952	28 040
Sex															
	Female	2428 (64%)	686 (68·9%)	300 (68·7%)	2028 (65·5%)	461 (67·9%)	2507 (38·1%)	1695 (37·9%)	1393 (35·9%)	1689 (64·5%)	1264 (66·4%)	1187 (64·5%)	1680 (62·5%)	2046 (62·7%)	19 364 (64·5%)
	Male	991 (36%)	289 (31·1%)	136 (31·3%)	972 (34·5%)	153 (32·1%)	1182 (61·9%)	768 (62·1%)	641 (64·1%)	753 (35·5%)	559 (33·6%)	560 (35·5%)	766 (37·5%)	906 (37·3%)	8676 (35·5%)
Median age, years (IQR)		43 (37–50)	38 (30–46)	40 (32–47)	36 (29–45)	38 (30–45)	39 (32–50)	40 (32–49)	36 (28–45)	40 (32–47)	38 (30–47)	36 (28–45)	37 (30–44)	41 (33–49)	38 (30–47)
Age group, years															
	15–19	40 (1·4%)	36 (3·6%)	15 (2·9%)	112 (3·6%)	31 (4·9%)	66 (2·0%)	70 (3·5%)	66 (4·9%)	112 (5·1%)	39 (2·8%)	65 (4·1%)	88 (4·4%)	99 (4·2%)	839 (3·9%)
	20–24	117 (3·0%)	87 (7·7%)	20 (4·5%)	226 (7·9%)	31 (3·5%)	183 (4·6%)	131 (5·1%)	179 (10·9%)	129 (5·6%)	129 (7·1%)	163 (10·1%)	196 (8·3%)	147 (5·5%)	1738 (7·5%)
	25–29	207 (6·6%)	116 (9·5%)	42 (10·2%)	400 (14·1%)	74 (10·0%)	346 (10·4%)	203 (8·6%)	228 (12·9%)	206 (8·2%)	205 (11·3%)	247 (14·9%)	280 (12·2%)	203 (7·7%)	2757 (11·1%)
	30–34	285 (8·3%)	160 (17·2%)	59 (14·9%)	531 (18·2%)	101 (16·0%)	544 (17·2%)	304 (13·3%)	291 (15·9%)	318 (13·3%)	275 (15·1%)	270 (16·2%)	407 (16·6%)	338 (12·5%)	3883 (15·1%)
	35–39	519 (16·0%)	131 (15·9%)	83 (17·5%)	503 (17·8%)	137 (21·0%)	553 (16·8%)	417 (15·4%)	328 (15·1%)	433 (17·6%)	311 (16·9%)	274 (16·0%)	402 (16·6%)	439 (14·9%)	4530 (16·1%)
	40–44	650 (19·5%)	151 (17·1%)	62 (16·5%)	357 (13·3%)	97 (17·9%)	519 (13·1%)	388 (15·3%)	279 (13·2%)	421 (17·2%)	287 (15·3%)	223 (12·9%)	454 (17·4%)	431 (16·8%)	4319 (15·3%)
	44–49	611 (18·3%)	106 (10·3%)	50 (11·2%)	292 (9·8%)	57 (12·1%)	410 (9·8%)	360 (13·7%)	232 (9·7%)	328 (12·9%)	202 (11·6%)	226 (11·9%)	284 (11·7%)	448 (13·8%)	3606 (11·8%)
	50–54	456 (12·1%)	86 (9·8%)	38 (8·2%)	219 (6·4%)	47 (8·2%)	336 (9·2%)	230 (10·2%)	137 (6·3%)	240 (11·1%)	147 (7·9%)	135 (6·8%)	214 (7·9%)	294 (9·5%)	2579 (8·2%)
	55–59	327 (8·9%)	50 (5·1%)	40 (8·2%)	148 (3·9%)	18 (3·4%)	280 (7·0%)	135 (5·7%)	118 (4·5%)	162 (5·8%)	107 (6·7%)	83 (4·7%)	121 (4·9%)	239 (6·7%)	1828 (5·6%)
	60–64	207 (6·0%)	52 (3·9%)	27 (5·8%)	120 (2·7%)	21 (2·9%)	217 (5·0%)	101 (3·7%)	84 (2·8%)	93 (3·2%)	69 (3·0%)	61 (2·3%)	0	178 (4·6%)	1230 (3·2%)
	65 and older	0	0	0	92 (2·2%)	0	235 (4·8%)	124 (5·4%)	92 (3·8%)	0	52 (2·4%)	0	0	136 (3·7%)	731 (2·3%)
Residence															
	Rural	1665 (38·9%)	523 (45·5%)	173 (31·3%)	2276 (70·5%)	0	1756 (44·3%)	1902 (75·9%)	1059 (55·9%)	1559 (50·5%)	1079 (54·5%)	1134 (65·6%)	1039 (41·6%)	2149 (69·5%)	14 558 (53·3%)
	Urban	1754 (61·1%)	452 (54·5%)	263 (68·7%)	724 (29·5%)	614 (100%)	1933 (55·7%)	561 (24·1%)	975 (44·1%)	883 (49·5%)	744 (45·5%)	613 (34·4%)	1407 (58·4%)	803 (30·5%)	13 482 (46·7%)
Wealth quintile															
	Lowest	978 (22·0%)	183 (12·0%)	77 (26·8%)	750 (22·5%)	103 (17·1%)	759 (18·4%)	205 (11·8%)	343 (14·2%)	850 (27·3%)	330 (16·1%)	326 (13·9%)	241 (9·9%)	775 (22·4%)	5920 (21·2%)
	Second	826 (24·8%)	277 (24·6%)	112 (27·4%)	651 (20·9%)	108 (18·2%)	822 (21·3%)	239 (12·7%)	376 (15·1%)	643 (24·4%)	349 (18·4%)	287 (15·8%)	302 (12·5%)	680 (22·0%)	5672 (20·3%)
	Middle	632 (22·1%)	199 (20·6%)	101 (19·0%)	660 (21·5%)	143 (22·9%)	803 (22·7%)	395 (20·9%)	526 (20·5%)	536 (24·2%)	497 (25·1%)	354 (21·6%)	520 (20·6%)	559 (20·6%)	5925 (21·2%)
	Fourth	586 (19·7%)	177 (21·2%)	93 (17·0%)	524 (19·7%)	147 (23·6%)	703 (20·5%)	551 (27·8%)	632 (25·8%)	312 (17·8%)	388 (23·1%)	428 (28·3%)	675 (27·6%)	475 (18·2%)	5691 (20·3%)
	Highest	397 (11·4%)	139 (21·6%)	53 (9·8%)	412 (15·5%)	113 (18·1%)	559 (17·1%)	642 (26·8%)	584 (24·4%)	101 (6·2%)	259 (17·2%)	352 (20·4%)	694 (29·4%)	463 (16·8%)	4768 (17·0%)
Treatment cascade															
	Not aware of HIV status	153 (4·8%)	441 (44·3%)	215 (50·4%)	345 (13·0%)	119 (21·0%)	331 (9·9%)	253 (11·7%)	502 (28·5%)	297 (14·0%)	678 (39·4%)	440 (26·6%)	648 (28·3%)	340 (13·2%)	4762 (25·8%)
	Aware but not on ART	54 (1·9%)	36 (3·9%)	17 (4·0%)	282 (9·7%)	14 (2·3%)	96 (2·8%)	41 (1·9%)	49 (2·6%)	75 (3·1%)	74 (3·8%)	120 (7·1%)	219 (9·2%)	70 (2·6%)	1147 (4·1%)
	On ART but not virally supressed	66 (2·0%)	102 (10·4%)	47 (12·1%)	200 (6·7%)	62 (9·5%)	263 (7·5%)	68 (2·7%)	133 (7·3%)	202 (7·2%)	132 (7·3%)	202 (10·8%)	160 (6·7%)	234 (8·2%)	1871 (7·6%)
	Virally suppressed	3139 (91·3%)	390 (41·5%)	157 (33·5%)	2168 (70·6%)	414 (67·2%)	2995 (79·8%)	2098 (83·8%)	1345 (61·6%)	1863 (75·6%)	890 (49·6%)	982 (55·5%)	1391 (55·9%)	2308 (76·9%)	20,140 (62·5%)
	Median CD4 count (IQR)	622 (469–810)	465 (310–657)	536 (353–803)	534 (361–730)	433 (278–594)	566 (381–776)	524 (367–715)	515 (340–717)	535 (363–710)	423 (278–603)	500 (336–677)	422 (276–577)	494 (333–684)	523 (323–717)
CD4 count, cells per mm^3^															
	<200	89 (3·7%)	124 (13·5%)	27 (6·1%)	219 (7·6%)	82 (14·1%)	226 (6·6%)	138 (6·1%)	158 (7·8%)	189 (7·4%)	257 (14·7%)	163 (9·0%)	330 (13·9%)	259 (9·3%)	2261 (9·8%)
	200–349	324 (11·1%)	187 (18·7%)	71 (18·2%)	461 (15·6%)	139 (21·7%)	519 (14·5%)	380 (16·5%)	371 (18·5%)	381 (16·5%)	385 (21·5%)	291 (17·6%)	576 (23·8%)	502 (18·0%)	4587 (18·7%)
	350–499	600 (19·2%)	207 (23·0%)	98 (22·5%)	646 (21·5%)	156 (28·1%)	725 (20·0%)	565 (23·5%)	440 (21·4%)	531 (21·4%)	458 (25·5%)	401 (23·3%)	661 (26·7%)	656 (23·2%)	6144 (23·4%)
	≥500	2406 (65·9%)	457 (44·8%)	240 (53·2%)	1674 (55·3%)	237 (36·1%)	2219 (58·9%)	1380 (54·0%)	1065 (52·3%)	1341 (54·6%)	723 (38·2%)	892 (50·2%)	879 (35·7%)	1535 (49·5%)	15 048 (48·0%)

ART=antiretroviral treatment. Percentages in parentheses represent weighted proportions. The number in the cell divided by the sample size does not necessarily represent the displayed number.

**Figure 1:**
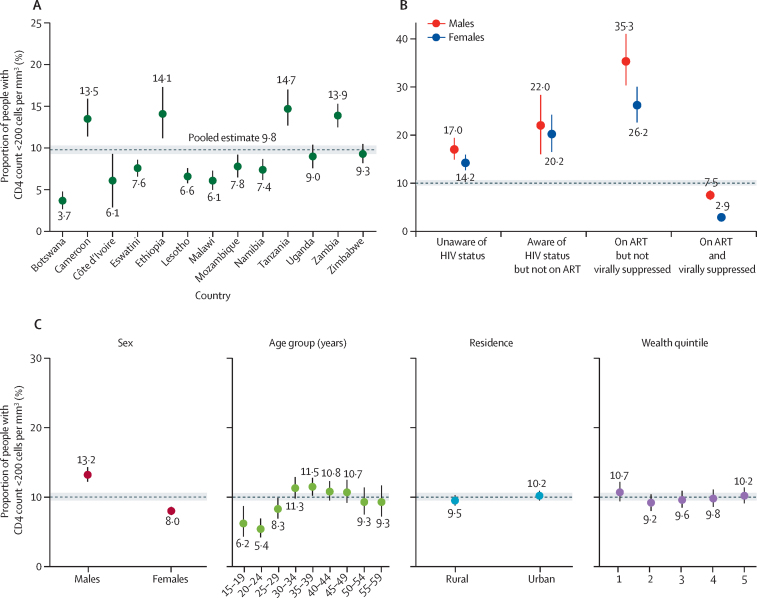
Proportion of people living with HIV with CD4 count below 200 cells per mm^3^ by country (A), the treatment cascade and sex (B), and various demographic and socioeconomic factors (C) ART=antiretroviral treatment.

Overall, 2261 (9·8% [95% CI 9·3–10·3]) people living with HIV had a CD4 count of less than 200 cells per mm^3^ (range: 3·7% in Botswana and Malawi to 14·7% in Tanzania; figure 1, table 2). Across the surveys, 18·7% (n=4587) had a CD4 count between 200 cells per mm^3^ and 349 cells per mm^3^, 23·4% (n=6144) between 350 and 499, and 48·0% (n=15 048) above or equal to 500 cells per mm^3^ (table 1). The median CD4 cell count per mm^3^ of people living with HIV was 523 (IQR 323–717) and differed across the HIV testing and treatment cascade (appendix p 7). The median CD4 cell count decreased with age, with stronger decreases among people living with HIV on all steps of the treatment cascade (appendix pp 2–4).

**Table 2:** Characteristics of individuals living with HIV with CD4 count below 200 cells per mm^3^ across the 13 included countries

		**Weighted proportion of AHD (%)**	**95% CI**	**n/N**
Overall		9·8	9·3–10·3	2261/28 040
Treatment cascade				
	Not aware of HIV status	15·4	14·1–16·7	729/4762
	Aware but not on ART	20·9	17·8–24·3	230/1147
	On ART therapy but not virally supressed	29·5	26·6–32·6	554/1871
	Virally suppressed	4·3	3·9–4·8	727/20 140
Sex				
	Female	8·0	7·4–8·6	1212/19 364
	Male	13·2	12·2–14·3	1049/8676
Age group, years				
	15–19	6·2	4·3–8·7	671/1458
	20–24	5·4	4·2–6·9	52/1687
	25–29	8·3	6·8–9·9	103/2650
	30–34	11·3	9·8–12·9	210/3721
	35–39	11·5	10·2–12·8	372/4481
	40–44	10·8	9·5–12·3	421/4361
	44–49	10·7	9·2–12·5	379/3705
	50–54	9·3	7·5–11·4	280/2683
	55–59	9·3	7·2–11·7	176/1883
	60–64	7·4	5·5–9·7	121/1264
	65 and older	12·3	8·5–17·0	87/758
Residence				
	Rural	9·5	8·8–10·3	12 390/25 779
	Urban	10·2	9·5–10·9	1169/13 559
Wealth quintile				
	Lowest	10·7	9·4–12·2	456/5952
	Second	9·2	8·0–10·4	406/5760
	Middle	9·6	8·5–10·9	463/5917
	Fourth	9·8	8·6–11·1	437/5587
	Highest	10·2	9·1–11·4	379/4660

Data are weighted proportion and confidence intervals. AHD=advanced HIV disease. ART=antiretroviral treatment.

Males were more likely to have AHD than females (13·2% [95% CI 12·2–14·3] *vs* 8·0% [7·4–8·6]; table 2). AHD prevalence was associated with older age, but not by type of place of residence (10·2% [95% CI 9·5–10·9] in urban *vs* 9·5% [8·8–10·3] in rural areas). There was no clear association between wealth quintile and AHD. AHD was strongly associated with the steps of the treatment and care cascade. The highest proportion of people with AHD was among people on ART who were not virally suppressed (29·5% [95% CI 26·6–32·6]), followed by people who knew their status but were not on ART (20·9% [17·8–24·3]) and people who did not know their status (15·4% [14·1–16·7]). People who were virally suppressed had the lowest AHD prevalence (4·3% [95% CI 3·9–4·8]). When data were disaggregated by sex, AHD prevalence was higher among males for every step on the cascade (figure 1). For example, males who were virally suppressed had an AHD prevalence of 7·5% (95% CI 6·5–8·5) compared with 2·9% (2·5–3·4) among females. The association between the steps of the treatment cascade and AHD was similar across all countries (appendix p 3).

**Figure 2:**
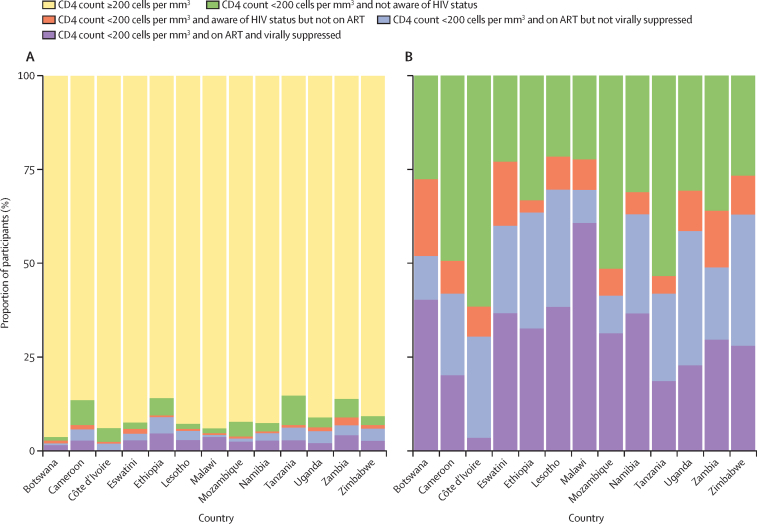
Stacked bar charts of the distribution of people living with HIV with CD4 count above and below 200 cells per mm^3^ by treatment cascade and country (A) Proportion of people with CD4 count ≥200 cells per mm^3^ and <200 cells per mm^3^ disaggregated by the treatment cascade. (B) Distribution of people on each step of the treatment cascade among all people with CD4 count <200 cells per mm^3^. ART=antiretroviral treatment.

In a mixed-effects logistic regression model that included the variable country as a random effect, male sex (odds ratio [OR] 1·88 [95% CI 1·71–2·06]) and age (OR per 5-year age increase 1·08 [1·06–1·10]) but not area of residence (OR 1·09 [0·98–1·20]) were associated with AHD independently from HIV treatment and viral suppression status (appendix p 11). Among people who were on ART but not virally suppressed, there was no association between the duration of ART and AHD (p=0·22; appendix p 12). Pooled across the 13 countries, 32·5% of people with AHD (n=727) had a suppressed viral load (appendix p 12) and proportions differed by country (figure 2).

Extrapolating the number of people with AHD to the sub-Saharan African region yielded 1·88 million individuals with AHD for 2022 (uncertainty interval [UI] 1·58–2·20), of whom 920 000 (UI 770 000–1·07 million) were female and 970 000 (UI 810 000–1·13 million) were male (table 3). This number corresponds with 7·7% of all people living with HIV having AHD. Among all people with AHD, 820 000 (43%) people had a suppressed viral load. This high level of viral suppression is larger than that seen across the PHIA surveys, highlighting the progress on access to ART and viral suppression in the years since the conduct of the PHIAs.

**Table 3:** Number of people living with HIV with CD4 cell count below 200 cells per mm^3^ disaggregated by sex and treatment cascade, extrapolated to the sub-Saharan African region

	**UNAIDS estimates**			**Number of people with AHD among treatment cascade groups (uncertainty interval)**			**Total number of people with AHD (uncertainty interval)**	**Proportion of people with AHD who were virally suppressed (uncertainty interval)**
	Number of people living with HIV*	Proportion on ART (uncertainty interval)*	Proportion virally suppressed (uncertainty interval)*	People not on ART	People not virally suppressed among those on ART	People who are virally suppressed		
All	24 200 000	83% (70–97)	77 (65–90)	670 000 (560 000–780 000)	390 000 (330 000–460 000)	820 000 (680 000–950 000)	1 880 000 (1 580 000–2 200 000)	43% (36–51)
Female	15 600 000	86% (71–98)	80 (74–90)	340 000 (280 000–390 000)	230 000 (190 000–260 000)	350 000 (300 000–410 000)	920 000 (770 000–1 070 000)	38% (32–45)
Male	8 600 000	78% (64–91)	73 (66–81)	340 000 (280 000–390 000)	160 000 (140 000–190 000)	470 000 (390 000–550 000)	970 000 (810 000–1 130 000)	48% (40–57)

Scenario if the 95–95–95 targets had already been met: 95% of all people living with HIV know their status and, of those who know their status, 95% are on treatment (90·25%); and of those on treatment 95% would have a suppressed viral load (85·74%): 1·61 million (incertainty interval 1·35–1·88 million**)** people would have advanced HIV disease, of whom 56% would have suppressed viral load. AHD=advanced HIV disease. ART=antiretroviral treatment.

* UNAIDS estimates from 2022.

If the 95–95–95 targets had already been achieved by 2022, there would have been an estimated 1·61 million (UI 1·35–1·88) people with AHD in the region (difference: 260 000 less than currently) and 56% of people with AHD would have been on ART and virally suppressed (table 3).

In sub-Saharan Africa, 25 (93%) of 27 reporting countries reported having fully adopted the recommendations from the 2021 WHO consolidated guidelines on HIV prevention, testing, treatment, service delivery, and monitoring. 17 (63%) of 27 countries in sub-Saharan Africa reported that these guideline recommendations are implemented in more than 95% of treatment sites. Only nine (35%) of 26 countries in sub-Saharan Africa had adopted all nine recommendations in their guidelines; however, 90% of countries had adopted at least six recommendations on tuberculosis preventive treatment, baseline CD4 testing to diagnose AHD, a molecular test for tuberculosis diagnosis, rapid ART initiation, and adapted adherence support. Recommendations on urine lateral flow lipoarabinomannan assay, cryptococcal antigen screening, and fluconazole pre-emptive therapy have not yet been implemented in more than 70% of countries in sub-Saharan Africa (appendix p 12).

## Discussion

Our analysis of household surveys from 13 countries and extrapolation of the proportion of people with a CD4 count below 200 cells per mm^3^ indicates that an estimated 1·88 million adults have AHD in sub-Saharan Africa. AHD was more common among males than females and increased with age, but the difference was not significant between urban and rural areas, nor was AHD associated with wealth. Substantial differences were seen across the treatment and care cascade, with the highest proportions of people with AHD among those on ART who were not virally suppressed. This finding was independent from reported ART duration, although no longitudinal data were available that would have allowed for an assessment of recent treatment interruptions.

The almost 30% of individuals on treatment with unsuppressed viral load who had AHD might be explained by recent ART initiation or re-initiation following a period of disengagement from care. Disengagement from care is common and is associated with progression to AHD globally.^15^ Some individuals who might have recently reinitiated ART after a period of treatment interruption might have achieved virological suppression before CD4 count recovery. A further proportion might be intermittently taking ART with periods of virological suppression, but these people might not be sufficiently sustained on ART to increase CD4 counts. A small number of individuals do not adequately reconstitute CD4 count despite sustained viral suppression on ART; this is more likely to occur among individuals who initiate ART with very low CD4 counts,^16^ and among individuals who have previously experienced treatment interruption.^17^ Reasons for disengaging from care are manifold and include structural and clinical barriers such as distance to health facility or waiting times, but also psychosocial factors like stigma and discrimination.^18^, ^19^ A further 21% of people who knew their HIV status but were not on ART, and 15% of those not aware of their HIV-positive status, had AHD. However, even among individuals with suppressed viral load, 4·3% had a CD4 count below 200 cells per mm^3^.

Other studies from South Africa and from regions outside sub-Saharan Africa have reported similar findings of continued high burden of advanced HIV disease.^15^, ^20^, ^21^ A review published in 2015 also found that HIV still represents a significant proportion of hospital admissions with a substantially elevated mortality in high prevalence countries;^22^ this finding was unchanged when the review was updated to 2023.^23^ Our findings highlight the importance of providing HIV services in ways that encourage clients to engage with services for earlier diagnosis and to remain in care, and to support the tracing and return to care for those who have previously disengaged from HIV services.

Across countries, men had a higher AHD prevalence and AHD prevalence also increased with older age, independent of HIV treatment status, which was consistent with previous studies.^6^, ^17^, ^18^ Men have been shown to test for HIV less, are diagnosed with HIV later, initiate treatment with longer delays, and generally have a lower ART coverage than women.^24^ Each of these factors contribute to the development of AHD and probably result in this sex differential. Additionally, for every 5-year increase in age, the odds of AHD increased by 8%. Although we could not assess longitudinal data in this study, our assessment of CD4 cell count by age highlighted a large difference in median CD4 cell count even for people with suppressed viral load, which might be due to a combination of ageing (with HIV) and low CD4 baseline cell counts at diagnosis or initiation of ART. Since immune recovery after ART initiation is stronger and faster if the baseline CD4 count is higher,^25^ the long-term implication of these results is poorer health outcomes and higher mortality for older age groups and people with low CD4 count at ART initiation. Furthermore, our findings demonstrate that AHD is prevalent not only in rural areas but equally also in urban settings, challenging the common assumption that health care and service provision are adequate in urban areas. This suggests that while rural areas certainly require attention, urban areas also need targeted interventions to address health outcomes effectively.

In 2022, approximately 540 000 adults died from AIDS-related conditions globally, 220 000 in eastern and southern Africa and 89 000 in western and central Africa. Although tuberculosis is still the number one cause of death among people with AHD, studies highlighted a large burden of cryptococcal meningitis and severe bacterial infections in patients admitted to hospital with AHD and a large burden of histoplasmosis in certain areas of the world.^26^, ^27^ WHO has published guidelines for the management of advanced HIV disease which include recommendations on a package of interventions aimed at reducing HIV-associated morbidity and mortality including CD4 count testing, tuberculosis diagnosis, tuberculosis preventive treatment, cryptococcal antigen screening, co-trimoxazole prophylaxis, rapid ART initiation, and enhanced adherence support.^1^, ^4^, ^5^ In the NCPI, most countries reported having adopted several items from the recommended AHD package in their national policies, which is encouraging progress. Policy adoption among the remaining countries and further expansion of their implementation to most ART sites are needed for diagnosis and treatment of conditions associated with AHD. Advancements in the scale and quality of case-based surveillance from routinely collected data could improve the timeliness and quality of monitoring of HIV-related indicators, including the incidence and prevalence of AHD and opportunistic infections.^28^ Such routine data collection would enable longitudinal analyses starting from HIV testing, and engagement in HIV care and treatment, as well as retrospective assessments of missed opportunities for HIV testing or the provision of the prevention package of AHD.

Our definition of AHD relied solely on CD4 cell count tests, as no additional clinical information was available in the PHIA surveys. This will have resulted in an underestimate of the number of people with AHD, but apart from underestimating the number of people with AHD because of pulmonary tuberculosis, we believe the effect is probably minimal. This is because most individuals with symptoms associated with AHD first have a drop of CD4 count less than 200 cells per mm^3^ and only subsequently develop diseases of WHO stage 3 or 4 HIV infection. Also, AHD assessment by clinical staging alone is only recommended in areas where CD4 count testing is not available and has been shown to have poor sensitivity.^29^ In addition, 10% of participants overall in the PHIA surveys did not have a CD4 count result, and this might have further resulted in an underestimate of the proportion of people with AHD. However, demographic characteristics of survey participants with and without CD4 test results were similar across several factors, apart from in females and those in rural areas having slightly lower rates of CD4 testing, so we do not anticipate that participants without a CD4 cell count result were considerably different to those with a test result. PHIA surveys were conducted between 2016 and 2021, and while we were able to account for advancements in the treatment cascade, we could not entirely account for the effect of the time between adoption of “treat all” and the conduct of the surveys. This means that the proportion of people who were diagnosed before an adoption of the “treat all” approach might differ between earlier and later surveys, which might have influenced the proportion of people with AHD, as generally, a lower CD4 count at ART initiation is associated with a weaker immune reconstitution. Additionally, we were not able to assess the route of transmission. The high prevalence of AHD among individuals aged 15–19 years raises the question of whether these individuals were infected vertically. However, as vertical transmission rates are slowly reducing, the effect on our estimates would probably have been marginal. We included data from countries with approximately 11 million of the 24·2 million estimated adults living with HIV. Although this represents a high proportion of adults living with HIV, it is important to note that some effects of advanced HIV disease can remain unobserved, particularly due to the under-representation of countries with low HIV prevalence.

Importantly, PHIA surveys are household surveys and do not include information on certain populations at higher risk of AHD, such as mobile populations or people who were admitted to hospital during the time of the conduct of the survey. Most people admitted to hospital with HIV are severely unwell, and are likely to have AHD. Studies have shown that the proportion of individuals with advanced HIV disease in hospital settings can be greater than 40%.^3^ Hence, AHD prevalence will be higher if individuals receiving care in health-care settings are accounted for.^30^ For these reasons, we consider our estimate of AHD to be conservative, and the lower bound of what could be expected in the region. Coverage of PHIA surveys is variable, with less than 6% of data included in these surveys coming from only two countries in West and Central Africa, a region where the delivery of HIV services has long been challenging.^31^ Finally, guideline adoption at a policy level does not provide information about the extent to which the services are available within a country.

A substantial number of people continue to develop AHD, even from our conservative estimates based on household surveys, which do not capture data from health facilities. A considerable proportion of people with AHD were on ART, some of whom had a suppressed viral load indicating that these individuals might have recently initiated ART or re-engaged in ART after treatment interruption. Our study highlights the need for urgent and innovative programmatic improvements in monitoring, prevention, and diagnosis of AHD to prevent adverse health outcomes in the context of well-established and maturing ART programmes.

### Contributors

## Data sharing

Data used in this manuscript are publicly available at https://phia-data.icap.columbia.edu/.

## Declaration of interests

We declare no competing interests.
