# Supplementary material for: Prevalence of advanced HIV disease in sub-Saharan Africa: a multi-country analysis of nationally representative household surveys
Source: Lancet Glob Health. 2025 Feb 26;13(3):e437–46. doi: 10.1016/S2214-109X(24)00538-2 (PMC11868778; doi:10.1016/S2214-109X(24)00538-2)
Supplement: Supplementary appendix [file mmc1.pdf]

THE LANCET
Global Health

Supplementary appendix

This appendix formed part of the original submission and has been peer reviewed. We post it as supplied by the authors.

Supplement to: Stelzle D, Rangaraj A, Jarvis JN, et al. Prevalence of advanced HIV disease in sub-Saharan Africa: a multi-country analysis of nationally representative household surveys. *Lancet Glob Health* 2025; **13:** e437–46.

SUPPLEMENT

High Prevalence of Advanced HIV Disease in Sub-Saharan Africa: An Analysis of National Representative Household Surveys

Dominik Stelzle MD[1], Ajay Rangaraj MSc PH[1], Jospeh N Jarvis MBBS[2,3], Nirina H Razakasoa MPH[1,4], George Perrin MD[4], Daniel Low-Beer PhD[1], Meg Doherty PhD[1], Nathan Ford DSc[1,5], Shona Dalal PhD[1]

1 Global HIV, Hepatitis and STIs Programmes, World Health Organization, Geneva, Switzerland
2 London School of Hygiene and Tropical Medicine, London, United Kingdom
3 Botswana Harvard Health Partnership, Gaborone, Botswana
4 Regional Office for Africa, World Health Organization, Brazzaville, Republic of the Congo
5 Centre for Integrated Data Epidemiological Research, University of Cape Town, Cape Town, South Africa

Figure S1. Density curves of CD4 count and age group, by cascade

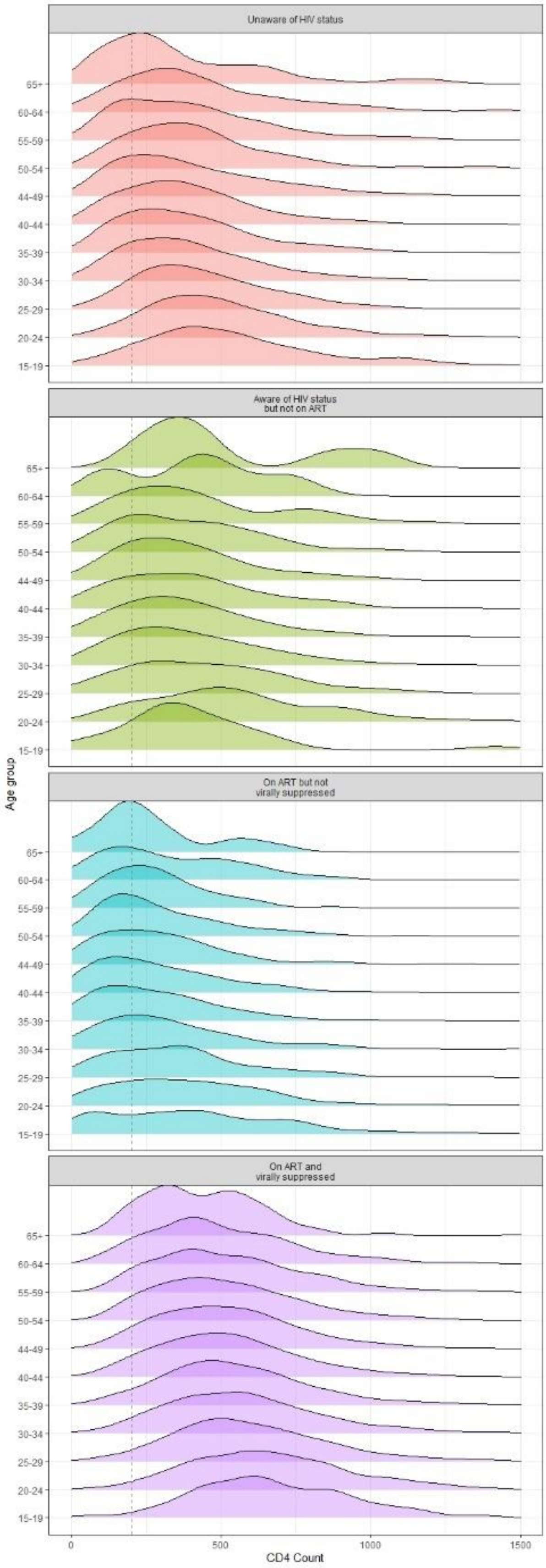

Figure S2. Density curves of CD4 count, by cascade and country

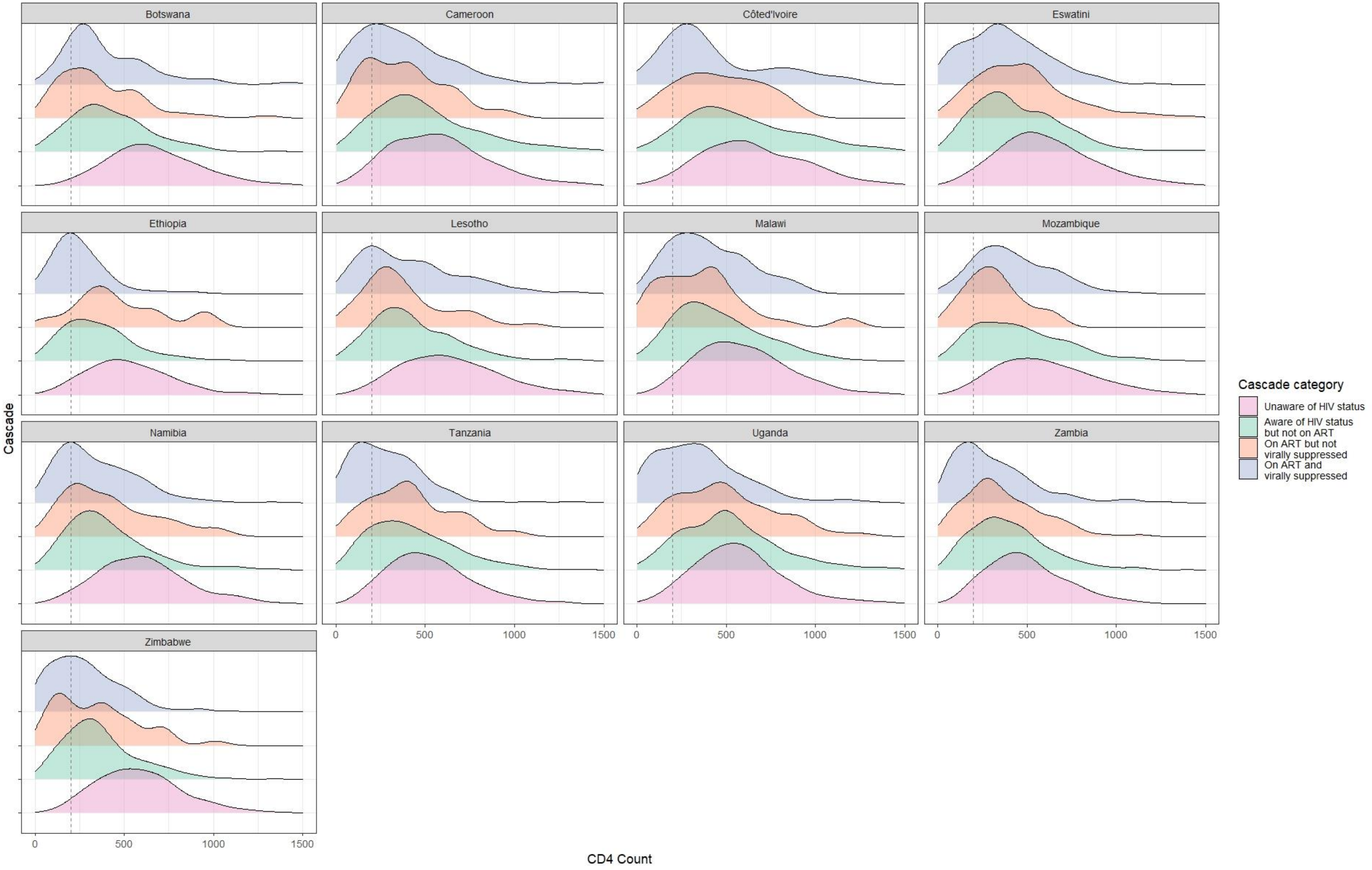

Figure S3. CD4 count by age, cascade and sex

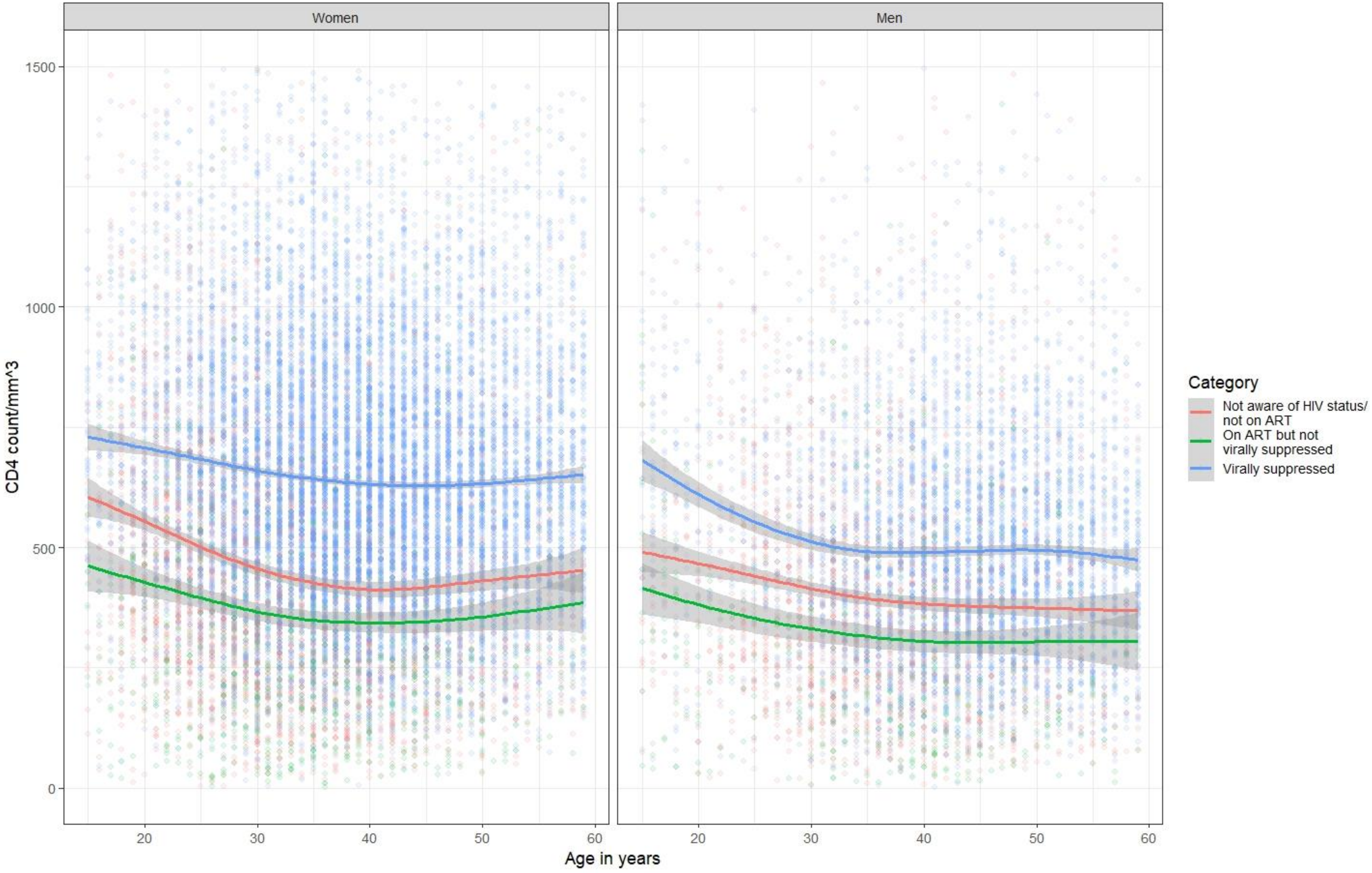

Table S1. Available PHIA datasets with CD4 count testing

	Year survey
Botswana	2021
Cameroon	2017
Cote d'Ivoire	2017
Eswatini	2016
Ethiopia	2017
Lesotho	2020
Malawi	2020
Mozambique	2021
Namibia	2017
Tanzania	2016
Uganda	2016
Zambia	2016
Zimbabwe	2020

Table S2. Differences between people with and without valid CD4 count test result

		**People with CD4 count**		**People without CD4 count**	
Sex	Women	19364	64·5	1802	70·1
	Men	8676	35·5	767	29·9
Age in years	Median (IQR)	38	30-47	39	30-48
Age group	15-19	839	3·9	96	3·7
	20-24	1738	7·5	170	6·6
	25-29	2757	11·1	288	11·2
	30-34	3883	15·1	395	15·4
	35-39	4530	16·1	354	13·8
	40-44	4319	15·3	391	15·2
	44-49	3606	11·8	302	11·8
	50-54	2579	8·2	267	10·4
	55-59	1828	5·6	191	7·4
	60-64	1230	3·2	112	4·4
	65+	731	2·3	3	0·1
Residence	Rural	14558	53·3	1563	60·8
	Urban	13482	46·7	1006	39·2

Table S3. Sex-disaggregated prevalence of AHD by the treatment cascade

Cascade step	AHD prevalence % (95%CI)	
	Men	Women
Not aware of HIV status or aware but not on ART[£]	17·7 (15·7–19·9)	15·0 (13·6–16·5)
Not aware of HIV status	*17·0 (14·9–19·4)*	*14·2 (12·7–15·9)*
Aware of HIV status but not on ART	*22·0 (16·0–28·3)*	*20·2 (16·5–24·2)*
On ART but not virally suppressed	35·3 (30·3–41·0)	26·2 (22·6–30·0)
On ART and virally suppressed	7·5 (6·5–8·5)	2·9 (2·5–3·4)

[£] For the estimation of the number of people with AHD, the first two steps of the cascade were collided.

Table S4. Characteristics of individuals with CD4 count below 200[£] by country

		Botswana			Cameroon			Cote d'Ivoire			Eswatini		
		Proportion	95% CI	n/N	Proportion	95% CI	n/N	Proportion	95% CI	n/N	Proportion	95% CI	n/N
Overall		3.7	(2.7-4.8)	89/3419	13·5	(11·4-15·9)	124/975	6·1	(2·9-9·3)	27/436	7·6	(6·6-8·6)	219/3000
Cascade	Not aware of HIV status	21.5	(11.9-34.1)	23/153	15·1	(11·4-19·3)	59/441	7·4	(3·6-13·4)	14/215	13·4	(10-17·5)	47/345
	Aware but not on ART	39.4	(17.9-64.4)	14/54	30·2	(17·6-45·6)	10/36	12·1	(1-41·3)	2/17	13·2	(9·8-17·4)	38/282
	On ART but not VS	22.3	(9.2-41.2)	11/66	28·4	(18·2-40·5)	30/102	13·6	(4·3-29·8)	8/47	26·3	(19·5-33·9)	50/200
	Virally suppressed	1.6	(1.1-2.3)	41/3139	6·5	(3·9-10·1)	24/390	0·6	(0·1-1·9)	3/157	3·9	(3·1-4·9)	83/2168
Sex	Women	2	(1.2-3.2)	36/2428	13	(10·3-16·1)	79/686	6·3	(2·6-12·2)	18/300	5·6	(4·6-6·6)	109/2028
	Men	6.8	(4.7-9.5)	53/991	14·7	(10-20·5)	45/289	5·7	(2·3-11·6)	9/136	11·4	(9·4-13·5)	110/972
Age group	15-19	0		0/40	2·5	(0·2-9·6)	2/36	0		0/15	10·3	(4·4-19·6)	84/185
	20-24	0		0/117	6·4	(1·8-15·7)	6/87	15	(0·2-64·9)	1/20	8·9	(5·3-13·8)	11/218
	25-29	3	(1.3-5.6)	9/207	13·4	(7-22·4)	14/116	0·4	(0-2·2)	1/42	6·2	(4-9)	19/394
	30-34	6.1	(2.3-12.8)	10/285	20·7	(14·9-27·7)	31/160	5·8	(0·9-18)	5/59	7·2	(4·7-10·4)	25/520
	35-39	3.5	(1.9-5.8)	11/519	16·8	(11-24)	21/131	8·3	(2-21)	5/83	9·2	(6·6-12·5)	36/493
	40-44	5.4	(3.2-8.5)	21/650	7·4	(3·5-13·4)	15/151	8·5	(1·9-22·6)	4/62	7·2	(4·6-10·5)	46/378
	44-49	5.2	(2.1-10.3)	18/611	17	(9·1-27·9)	14/106	5·4	(1·3-14·1)	4/50	6·8	(3·8-10·9)	25/299
	50-54	1.8	(0.7-3.7)	11/456	16·2	(7·9-28·2)	12/86	3	(0·3-11·3)	2/38	5·7	(2·7-10·3)	18/226
	55-59	1.7	(0.5-4.2)	6/327	8	(3·3-15·9)	3/50	5·9	(1-17·9)	3/40	6·6	(3-12·2)	11/150
	60-64	1.4	(0.2-5.2)	3/207	10·8	(3·5-23·8)	6/52	5·5	(0·2-24·7)	2/27	9·5	(4·6-17)	9/118
	65+	0	(0-0)	/0									
Residence	Rural	3.2	(2.1-4.6)	1706/3330	15·1	(11·4-19·3)	69/523	5·4	(2·1-11)	9/173	7·2	(6·1-8·4)	156/2276
	Urban	4.1	(2.6-6)	41/1747	30·2	(17·6-45·6)	55/452	6·4	(2·8-12·4)	18/263	8·5	(6·9-10·4)	63/724
Wealth quintile	Lowest	3.8	(2.2-6.2)	26/978	20·5	(13·7-28·8)	32/183	6·7	(1·8-16·4)	5/77	7·7	(5·9-9·8)	55/750
	Second	4.5	(2.7-7)	22/826	11	(7-16·3)	32/277	5·9	(1·5-15·1)	7/112	7·3	(5·2-10)	42/651
	Middle	1.8	(0.8-3.5)	10/632	14·7	(9·7-21·2)	26/199	6	(2-13·5)	8/101	8·1	(6-10·6)	51/660
	Fourth	4.3	(1.4-10)	19/586	11·7	(6·9-18·1)	16/177	9·1	(3·1-19·4)	7/93	8·1	(6·4-9·9)	42/524
	Highest	4.7	(1.6-10.4)	12/397	13·1	(7·8-20·1)	18/139	0	(0-0)	0/53	6·2	(4·2-8·8)	27/412

Note: ART: antiretroviral treatment; VS: virally suppressed; IQR: interquartile range, CI: confidence interval; £: weighted proportion and confidence intervals.

Table S4. – cont'd

		Ethiopia			Lesotho			Malawi		
		Proportion	95% CI	n/N	Proportion	95% CI	n/N	Proportion	95% CI	n/N
Overall		14·1	(11·2-17·3)	82/614	6·6	(5·8-7·5)	226/3689	6·1	(5-7·2)	138/2463
Cascade	Not aware of HIV status	22	(14·4-31·4)	28/119	14·5	(10·3-19·6)	48/331	11·7	(7·8-16·5)	32/253
	Aware but not on ART	19·7	(1·9-58·2)	2/14	20·9	(13·3-30·4)	20/96	26·4	(13·2-43·8)	11/41
	On ART but not VS	45	(31·5-59·1)	28/62	27·7	(21·5-34·5)	69/263	19·7	(10·6-31·8)	13/68
	Virally suppressed	6·7	(4-10·5)	23/414	3·2	(2·5-3·9)	89/2995	4·4	(3·4-5·6)	82/2098
Sex	Women	12·8	(9·6-16·5)	56/461	4·5	(3·7-5·4)	112/2507	4·1	(3·1-5·3)	66/1695
	Men	16·9	(10·6-24·9)	26/153	10·1	(8·4-11·9)	114/1182	9·2	(7·1-11·8)	72/768
Age group	15-19	12	(3·7-26·8)	4/31	3·1	(0·4-10·7)	226/290	7	(2·3-15·5)	117/182
	20-24	14·9	(3·9-34·8)	5/31	5·7	(2·5-11·1)	2/176	2·2	(0·3-7·5)	5/134
	25-29	12·3	(4·2-26·5)	10/74	6·4	(4-9·6)	9/333	4·7	(2·2-8·7)	2/194
	30-34	20·1	(12-30·4)	19/101	8·4	(5·8-11·5)	22/525	4·7	(2·4-8·1)	11/301
	35-39	13·3	(7·5-21·3)	15/137	7·6	(5·4-10·3)	41/556	6·9	(4·3-10·4)	14/407
	40-44	9·6	(3·6-19·8)	8/97	7·6	(5·5-10·2)	38/520	7·7	(4·9-11·3)	24/383
	44-49	14·2	(6·7-25·3)	7/57	5·3	(3·3-8)	37/425	6	(3·1-10·3)	29/370
	50-54	12·8	(4·8-25·8)	8/47	5·6	(3·2-9)	22/339	5	(2·2-9·5)	19/240
	55-59	30·2	(9·8-58·6)	5/18	4	(2·1-6·7)	19/288	9·5	(5-16)	9/133
	60-64	7·4	(0·4-30·7)	1/21	8·4	(4·7-13·6)	11/212	7·1	(2·4-15·6)	11/105
	65+				4·1	(1·9-7·5)	16/242	5·6	(1·9-12·4)	7/124
Residence	Rural	0			6·1	(4·9-7·6)	1803/3463	5·6	(4·4-7)	523/2325
	Urban	14·1	(11·2-17·3)	82/614	7	(5·9-8·3)	96/1899	7·5	(5·4-10·1)	100/623
Wealth quintile	Lowest	24	(14·1-36·4)	21/97	6·5	(4·4-9·2)	42/759	7·3	(4·4-11·4)	24/343
	Second	14·4	(6·8-25·7)	15/111	7·3	(5·7-9·1)	57/822	6	(3·6-9·3)	19/376
	Middle	13·4	(7·2-22)	18/142	7·1	(5·1-9·6)	53/803	5·8	(3·8-8·4)	29/526
	Fourth	11·3	(5·8-19·2)	17/141	5·9	(4·1-8·1)	39/703	5·4	(3·4-8·1)	33/632
	Highest	8·9	(3·8-17·1)	11/102	6·3	(4·3-8·9)	33/559	6·3	(4·3-8·8)	33/584

Note: ART: antiretroviral treatment; VS virally suppressed; IQR: interquartile range, CI: confidence interval; £: weighted proportion and confidence intervals.

Table S4. – cont'd

		Mozambique			Namibia			Tanzania		
		Proportion	95% CI	n/N	Proportion	95% CI	n/N	Proportion	95% CI	n/N
Overall		7·8	(6·5-9·1)	158/2034	7·4	(6·2-8·7)	189/2442	14·7	(12·7-17)	257/1823
Cascade	Not aware of HIV status	14·1	(11·1-17·6)	77/502	16·5	(11·6-22·4)	47/297	19·8	(16·5-23·4)	125/678
	Aware but not on ART	21·6	(10·5-36·9)	11/49	14	(6·2-25·8)	14/75	17·9	(8·6-31·1)	15/74
	On ART but not VS	10·7	(6·4-16·5)	15/133	27·2	(21·1-34·1)	61/202	46·6	(34·7-58·9)	53/132
	Virally suppressed	4	(3-5·1)	55/1345	3·6	(2·7-4·7)	67/1863	5·5	(3·7-7·8)	51/890
Sex	Women	6·3	(4·9-7·9)	86/1393	4·9	(3·8-6·2)	94/1689	12·7	(10·5-15·2)	154/1264
	Men	10·5	(8·1-13·3)	72/641	12	(9·7-14·7)	95/753	18·8	(14·9-23·2)	103/559
Age group	15-19	5·6	(2·1-11·9)	79/140	1·2	(0-6·2)	1/112	6·9	(0·6-25·1)	2/39
	20-24	2·3	(0·6-5·9)	5/179	4·6	(1·8-9·5)	6/129	6·5	(2·4-13·7)	9/129
	25-29	6·3	(3·8-9·9)	5/217	6·2	(2-14)	11/206	14·7	(8·9-22·4)	30/205
	30-34	8·9	(5·2-14·1)	16/286	7·4	(4·3-11·7)	27/318	13·9	(9·1-19·9)	38/275
	35-39	11·2	(8-15·2)	21/312	10·3	(7-14·6)	45/433	12·7	(9·1-17)	42/311
	40-44	8·5	(5·6-12·2)	37/292	9·3	(6·5-12·6)	42/421	17·3	(11·8-24)	43/287
	44-49	7·5	(3·9-12·6)	24/239	8	(5·2-11·7)	24/328	21·3	(14·8-29·2)	41/202
	50-54	6·6	(2·1-15)	17/148	5·8	(3·2-9·6)	17/240	19·6	(12·2-29)	26/147
	55-59	6·8	(2·9-13·1)	6/116	5·1	(2·4-9·6)	9/162	11·5	(4·3-23·6)	12/107
	60-64	6·9	(2·1-15·8)	8/86	6·7	(2·6-13·7)	7/93	8·5	(2·9-18·6)	8/69
	65+	15·2	(7·8-25·5)	6/85				20·8	(7·2-41·8)	6/52
Residence	Rural	7·7	(5·8-9·8)	905/1876	7·7	(6·4-9·3)	118/1559	15·7	(12·8-19)	157/1079
	Urban	8	(6·3-9·9)	88/993	7·1	(5·2-9·3)	71/883	13·6	(11·1-16·4)	100/744
Wealth quintile	Lowest	6·5	(3·7-10·6)	18/205	7·6	(5·9-9·6)	62/850	14·8	(10-20·8)	50/330
	Second	7·4	(4·5-11·5)	19/239	8·9	(6·3-12·2)	58/643	13	(8·7-18·4)	43/349
	Middle	8·5	(5·5-12·5)	35/395	7·8	(5·7-10·3)	49/536	13·8	(10·2-18·1)	72/497
	Fourth	6·7	(4·5-9·4)	36/551	5·6	(3·2-8·9)	16/312	18·8	(13·8-24·7)	61/388
	Highest	9·1	(6·8-12)	50/642	4·4	(1-12)	4/101	12·5	(8·8-17)	31/259

Note: ART: antiretroviral treatment; VS: virally suppressed; IQR: interquartile range, CI: confidence interval; £: weighted proportion and confidence intervals.

Table S4. – cont'd

		Uganda			Zambia			Zimbabwe		
		Proportion	95% CI	n/N	Proportion	95% CI	n/N	Proportion	95% CI	n/N
Overall		9	(7·6-10·4)	163/1747	13·9	(12·5-15·3)	330/2446	9·3	(8·2-10·5)	259/2952
Cascade	Not aware of HIV status	10·3	(7·7-13·5)	44/440	17·7	(15-20·6)	119/648	18·9	(15·1-23·1)	66/340
	Aware but not on ART	13·7	(8-21·2)	19/120	22·8	(17·4-29·1)	49/219	36·6	(23·7-51·1)	25/70
	On ART but not VS	29·6	(22·8-37·1)	59/202	39·8	(32·5-47·5)	62/160	39·8	(32·5-47·4)	95/234
	Virally suppressed	3·7	(2·7-4·9)	41/982	7·3	(5·9-9·1)	95/1391	3·4	(2·6-4·4)	73/2308
Sex	Women	7·7	(6·5-9·2)	93/1187	10·6	(9-12·3)	181/1680	5·9	(4·8-7·1)	128/2046
	Men	11·2	(8·5-14·3)	70/560	19·4	(16·8-22·3)	149/766	15	(12·6-17·7)	131/906
Age group	15-19	5·3	(1·4-13·5)	4/65	9·4	(4·3-17·3)	8/88	6·4	(2·2-13·9)	8/99
	20-24	3·8	(1·4-8·3)	7/163	7·6	(4·2-12·4)	16/196	11·8	(7·3-17·8)	18/147
	25-29	6·8	(3·9-10·8)	18/247	10·4	(6·8-14·9)	29/280	7·1	(3·9-11·7)	14/203
	30-34	10·1	(6·5-14·6)	29/270	16·8	(13·2-20·9)	65/407	10·6	(7·4-14·7)	36/338
	35-39	12·9	(9-17·6)	35/274	15·6	(12·1-19·6)	59/402	10·9	(7·8-14·7)	43/439
	40-44	12	(7·8-17·5)	26/223	16·8	(13·3-20·8)	71/454	8·7	(6-12·1)	34/431
	44-49	8·3	(5·1-12·8)	18/226	14·6	(10·6-19·5)	42/284	9	(6·1-12·7)	36/448
	50-54	6·1	(2·3-12·8)	9/135	11·8	(7·3-17·9)	23/214	7·9	(4·5-12·7)	23/294
	55-59	7·3	(2·6-15·7)	6/83	12·9	(7·3-20·6)	17/121	11·7	(7·7-16·8)	21/239
	60-64	14·7	(7·3-25·4)	11/61				4·9	(2·1-9·3)	9/178
	65+							10·9	(6·6-16·8)	17/136
Residence	Rural	8·6	(6·9-10·5)	101/1134	14·7	(12·7-16·9)	153/1039	8·7	(7·5-10·1)	177/2149
	Urban	9·7	(7·3-12·4)	62/613	13·3	(11·5-15·3)	177/1407	10·6	(8·3-13·4)	82/803
Wealth quintile	Lowest	9·7	(6·6-13·6)	27/326	20·3	(15·6-25·7)	49/241	9·4	(7·1-12)	68/775
	Second	7·7	(4·9-11·3)	24/287	13·3	(9·7-17·7)	42/302	8·6	(6·3-11·4)	52/680
	Middle	7·9	(5·6-10·8)	31/354	13·8	(10·8-17·3)	68/520	7·7	(5·5-10·3)	41/559
	Fourth	8·1	(5·4-11·5)	40/428	13·3	(10·9-16·1)	90/675	9·2	(6·6-12·4)	43/475
	Highest	11·7	(8·3-15·9)	41/352	12·7	(9·9-15·9)	80/694	12·2	(9·4-15·6)	55/463

Note: ART: antiretroviral treatment; VS: virally suppressed; IQR: interquartile range, CI: confidence interval; £: weighted proportion and confidence intervals.

Table S5. Median CD4 count (IQR) of HIV by the treatment cascade, by country

	PLHIV				
	Not aware of HIV status	Aware of HIV status but not on ART	On ART but not virally suppressed	On ART and virally suppressed	Total
Botswana	384 (260–588)	307 (194–514)	311 (250–534)	641 (492–828)	622 (469–810)
Cameroon	441 (306–657)	388 (184–520)	335 (173–491)	560 (375–739)	465 (310–657)
Côte d'Ivoire	526 (359–785)	414 (275–617)	331 (242–590)	610 (449–864)	536 (353–803)
Eswatini	401 (267–610)	449 (287–611)	348 (205–515)	577 (423–768)	534 (361–730)
Ethiopia	322 (210–471)	415 (315–626)	212 (160–323)	501 (370–673)	433 (278–594)
Lesotho	393 (265–583)	326 (226–492)	372 (193–580)	614 (433–815)	566 (381–776)
Malawi	413 (270–616)	366 (189–452)	380 (234–558)	561 (400–745)	524 (367–715)
Mozambique	419 (250-610)	311 (208-419)	380 (262-567)	575 (403-775)	515 (340–717)
Namibia	355 (241–518)	376 (226–589)	285 (178–485)	591 (429–753)	535 (363–710)
Tanzania	388 (241–578)	401 (231–561)	260 (132–401)	492 (358–661)	423 (278–603)
Uganda	490 (319–677)	463 (285–687)	323 (171–470)	550 (398–703)	500 (336–677)
Zambia	369 (246–520)	326 (219–497)	244 (150–423)	471 (337–630)	422 (276–577)
Zimbabwe	331 (221–474)	327 (155–502)	254 (140–387)	567 (412–729)	494 (333–684)
**Total**	**401 (260–588)**	**307 (233–554)**	**309 (177–484)**	**578 (415–828)**	**523 (323–717)**

PLHIV People living with HIV; IQR Interquartile range

Table S6. Results of the mixed effects logistic regression model of factors associated with AHD (with country included as random effect)

		Odds Ratio (95%CI)	p-values
Sex	Men	1·88 (1·71–2·06)	<0·001
Age	Per 5 years	1·08 (1·06–1·10)	<0·001
Residence	Urban	1·09 (0·98–1·20)	0·11
Cascade	Not aware of status	4·53 (4·03–5·10)	<0·001
	Aware of status but not on ART	6·59 (5·56–7·81)	<0·001
	On ART but not virally suppressed	11·46 (10·06–13·05)	<0·001
	On ART and virally suppressed	Reference	

Table S7. Self-reported ART duration and AHD (unweighted) among people who are on ART but not virally suppressed

ART duration	No AHD	AHD	Proportion AHD
>24 months	395	207	34·4%
12–24 months	73	27	27·0%
<12 months	128	54	29·7%
Self-reportedly not on ART	109	31	22·1%

p-value for association of AHD with duration on ART (excluding people who self-reportedly were not on ART): p=0·22

Table S8. Distribution of all people with AHD across the treatment cascade (n=2240)

Disaggregation of all people with AHD	Proportion
Not aware of HIV status	32·5
Aware of HIV status but not on ART	10·3
On ART but not virally suppressed	24·7
On ART and virally suppressed	32·5

Table S9. Components of the package of advanced HIV disease interventions in the national policy on ART for adults, adolescents and children; answers reported through the 2023 National Commitments and Policy Instrument.

	Sub-Saharan Africa n (%)
Adoption of AHD recommendations	27
Yes, fully adopted	25/27 (93)
Yes, partially adopted	2/27 (7)
No	0/27 (0)
Implementation of AHD recommendations in	27
>95% of treatment sites	17/27 (63)
50 to 95% of treatment sites	2/27 (7)
<50% of treatment sites	8/27 (30)
Other	0/27 (0)
Number of countries reporting on specific recommendations	26
Baseline CD4 test to diagnose advanced HIV disease	24/26 (92)
Molecular diagnostic tests for TB diagnosis	24/26 (92)
Urine LF-LAM for TB diagnosis	17/26 (65)
Cryptococcal antigen (CrAg) screening	17/26 (65)
Co-trimoxazole prophylaxis	23/26 (88)
TB preventive treatment	25/26 (96)
Fluconazole empirical prophylaxis	18/26 (69)
Fluconazole pre-emptive therapy	16/26 (62)
Rapid antiretroviral therapy initiation	24/26 (92)
Adapted adherence support	24/26 (92)
All above components included	9/26 (35)

NCPI National Commitments and Policy Instrument
